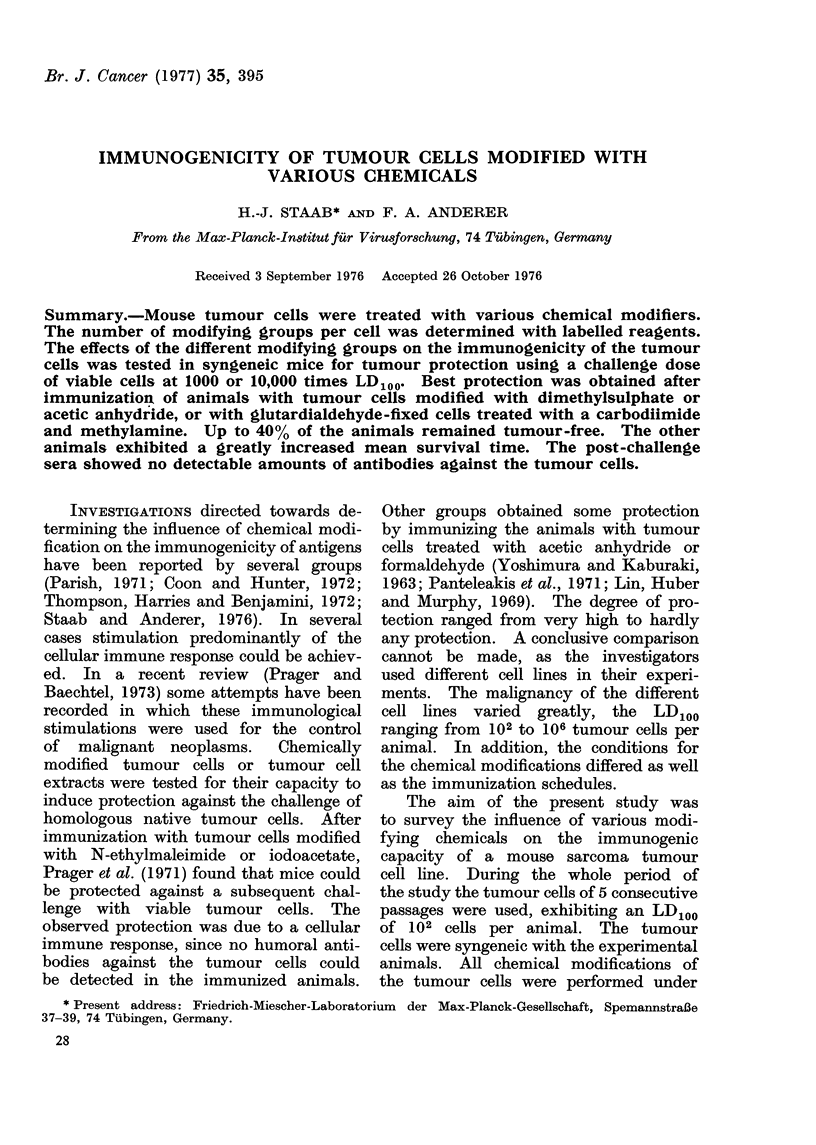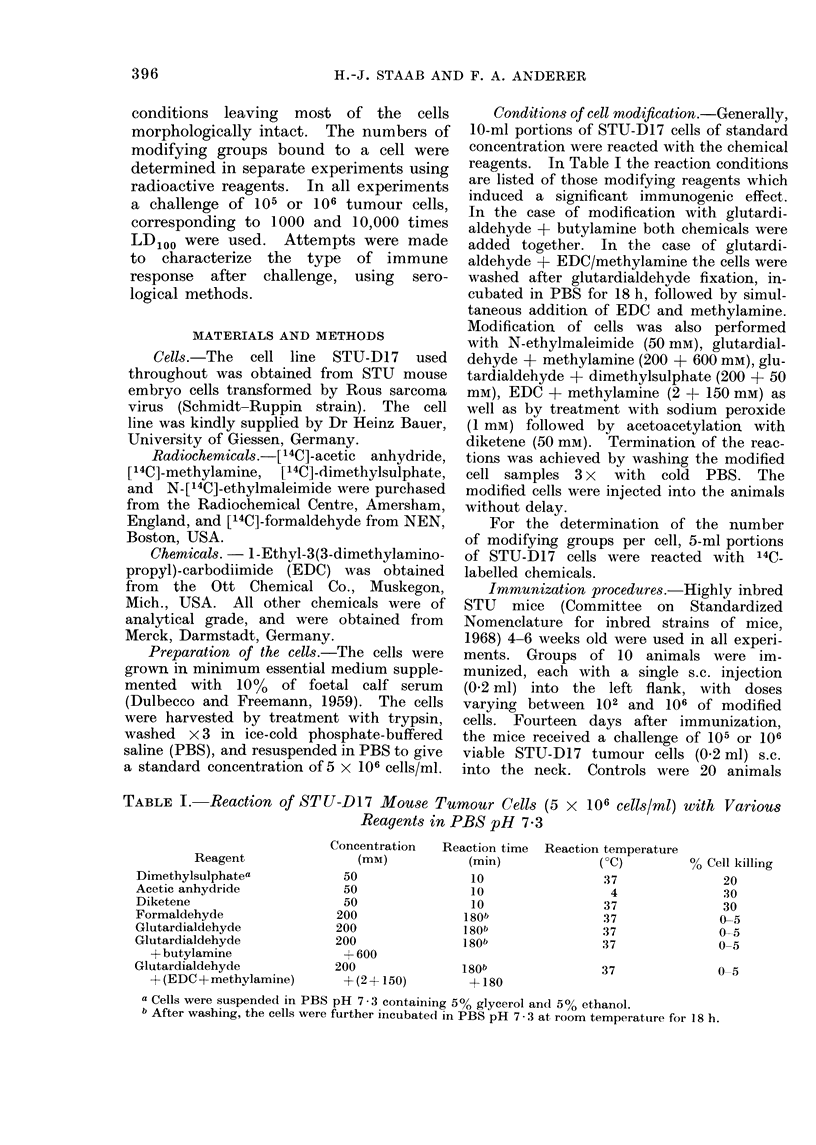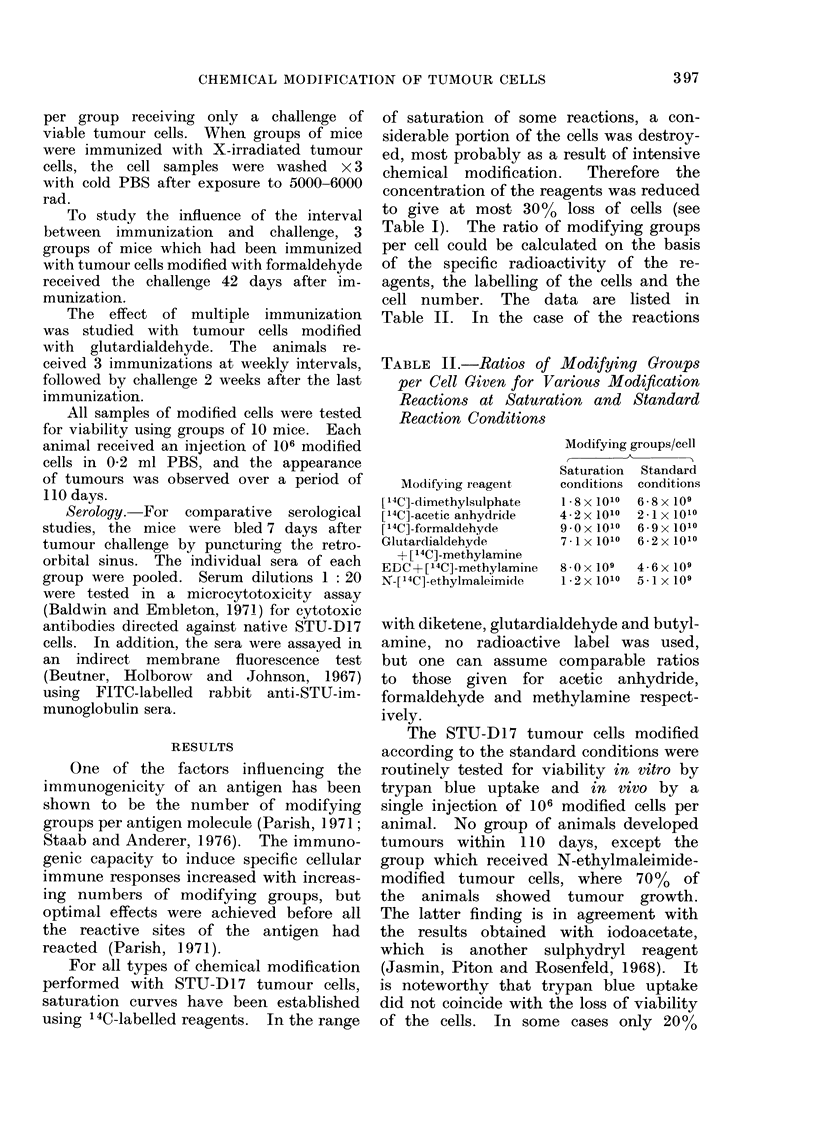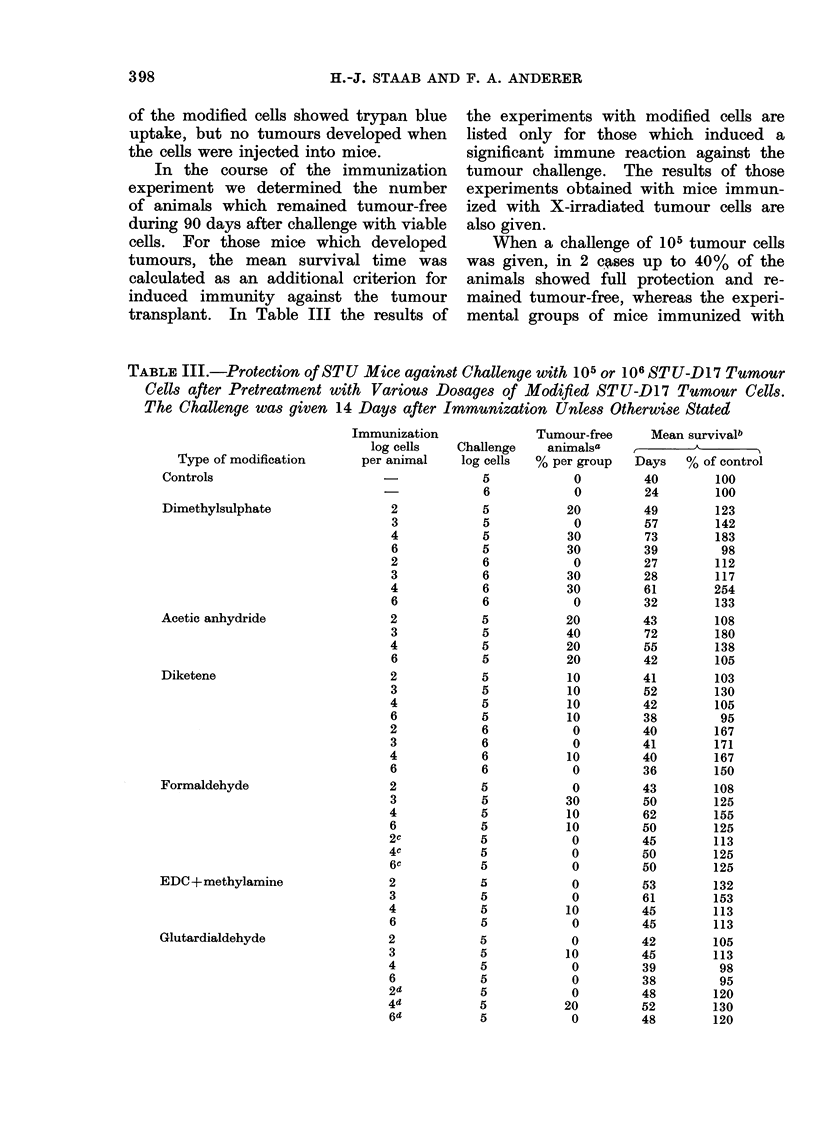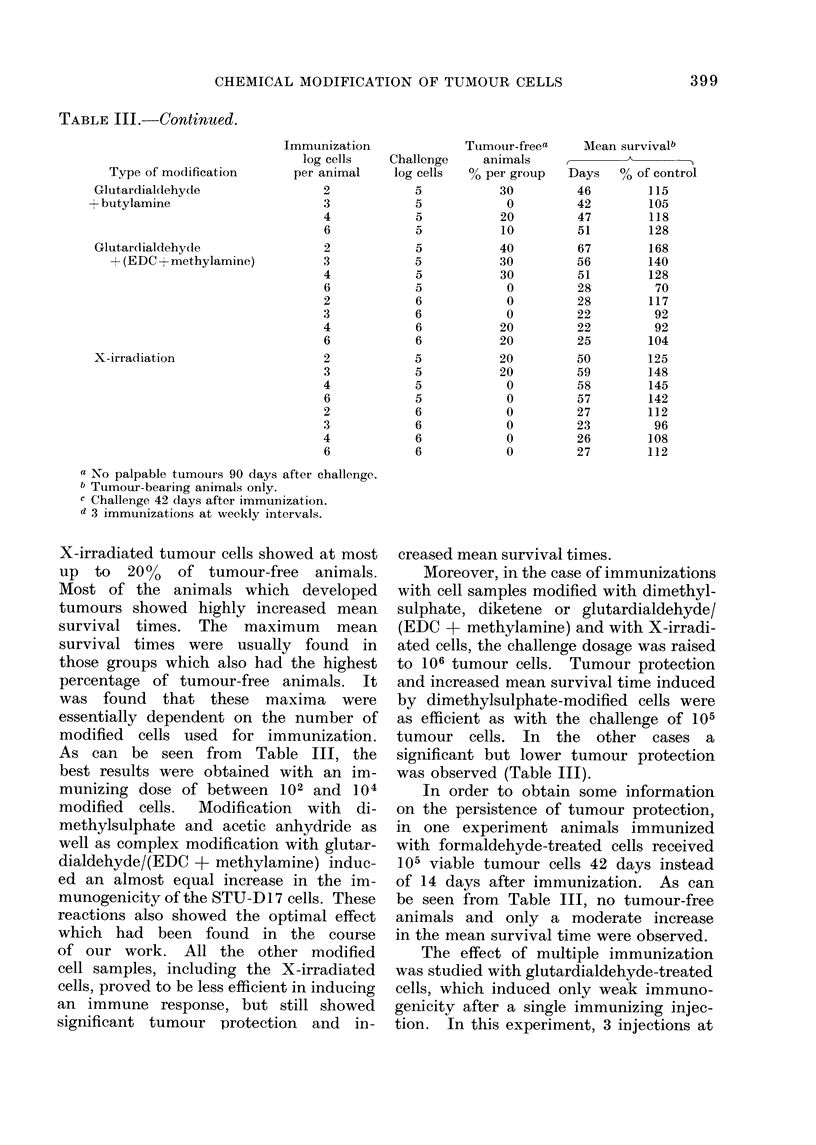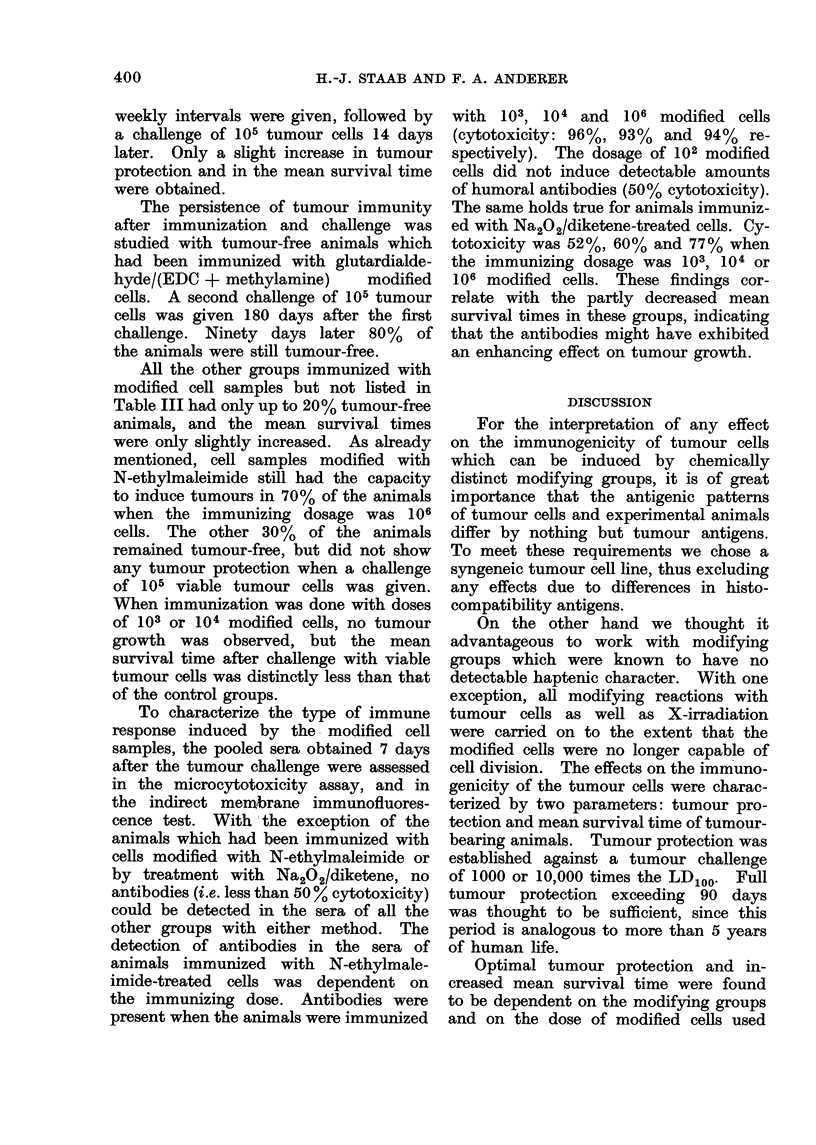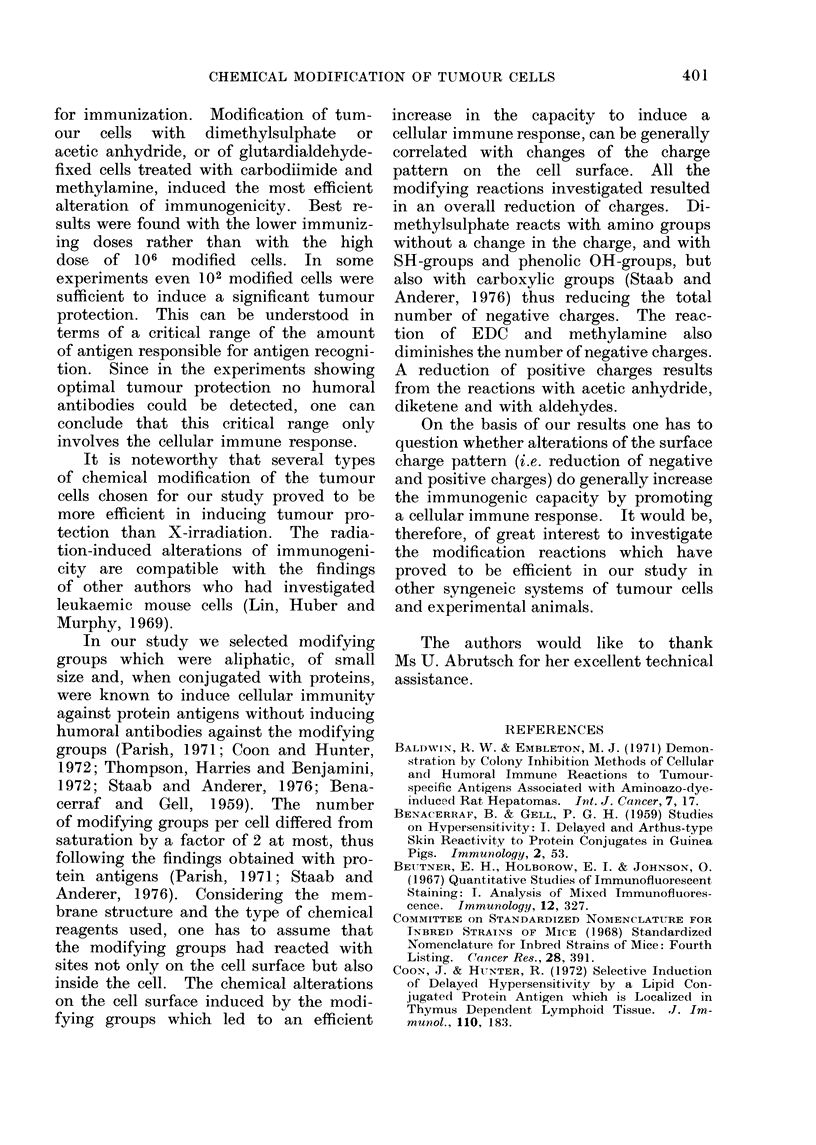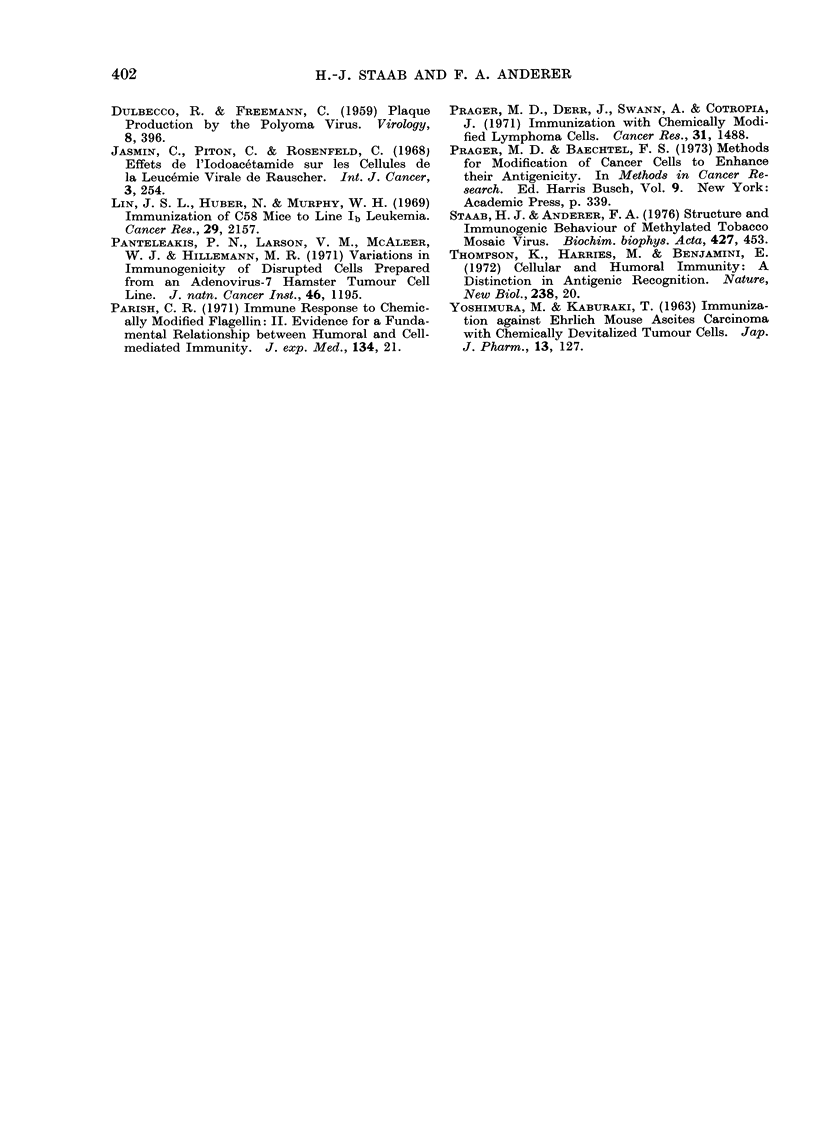# Immunogenicity of tumour cells modified with various chemicals.

**DOI:** 10.1038/bjc.1977.60

**Published:** 1977-04

**Authors:** H. J. Staab, F. A. Anderer

## Abstract

Mouse tumour cells were treated with various chemical modifiers. The number of modifying groups per cell was determined with labelled reagents. The effects of the different modifying groups on the immunogenicity of the tumour cells was tested in syngeneic mice for tumour protection using a challenge dose of viable cells at 1000 or 10,000 time LD100. Best protection was obtained after immunization of animals with tumour cells modified with dimethylsulphate or acetic anhydride, or with glutardialdehyde-fixed cells treated with a carbodiimide and methylamine. Up to 40% of the animals remained tumour-free. The other animals exhibited a greatly increased mean survival time. The post-challenge sera showed no detectable amounts of antibodies against the tumour cells.


*Br. J. Cancer* (1977) **35**, 395

### IMMUNOGENICITY OF TUMOUR CELLS MODIFIED WITH VARIOUS CHEMICALS

H.-J. STAAB* AND F. A. ANDERER

*From the Max-Planck-Institut für Virusforschung, 74 Tübingen, Germany*

Received 3 September 1976 Accepted 26 October 1976

**Summary.—Mouse tumour cells were treated with various chemical modifiers. The number of modifying groups per cell was determined with labelled reagents. The effects of the different modifying groups on the immunogenicity of the tumour cells was tested in syngeneic mice for tumour protection using a challenge dose of viable cells at 1000 or 10,000 times $LD_{100}$. Best protection was obtained after immunization of animals with tumour cells modified with dimethylsulphate or acetic anhydride, or with glutardialdehyde-fixed cells treated with a carbodiimide and methylamine. Up to 40% of the animals remained tumour-free. The other animals exhibited a greatly increased mean survival time. The post-challenge sera showed no detectable amounts of antibodies against the tumour cells.**

INVESTIGATIONS directed towards determining the influence of chemical modification on the immunogenicity of antigens have been reported by several groups (Parish, 1971; Coon and Hunter, 1972; Thompson, Harries and Benjamini, 1972; Staab and Anderer, 1976). In several cases stimulation predominantly of the cellular immune response could be achieved. In a recent review (Prager and Baechtel, 1973) some attempts have been recorded in which these immunological stimulations were used for the control of malignant neoplasms. Chemically modified tumour cells or tumour cell extracts were tested for their capacity to induce protection against the challenge of homologous native tumour cells. After immunization with tumour cells modified with N-ethylmaleimide or iodoacetate, Prager *et al.* (1971) found that mice could be protected against a subsequent challenge with viable tumour cells. The observed protection was due to a cellular immune response, since no humoral antibodies against the tumour cells could be detected in the immunized animals. Other groups obtained some protection by immunizing the animals with tumour cells treated with acetic anhydride or formaldehyde (Yoshimura and Kaburaki, 1963; Panteleakis *et al.*, 1971; Lin, Huber and Murphy, 1969). The degree of protection ranged from very high to hardly any protection. A conclusive comparison cannot be made, as the investigators used different cell lines in their experiments. The malignancy of the different cell lines varied greatly, the $LD_{100}$ ranging from $10^2$ to $10^6$ tumour cells per animal. In addition, the conditions for the chemical modifications differed as well as the immunization schedules.

The aim of the present study was to survey the influence of various modifying chemicals on the immunogenic capacity of a mouse sarcoma tumour cell line. During the whole period of the study the tumour cells of 5 consecutive passages were used, exhibiting an $LD_{100}$ of $10^2$ cells per animal. The tumour cells were syngeneic with the experimental animals. All chemical modifications of the tumour cells were performed under

* Present address: Friedrich-Miescher-Laboratorium der Max-Planck-Gesellschaft, Spemannstraße 37–39, 74 Tübingen, Germany.

conditions leaving most of the cells morphologically intact. The numbers of modifying groups bound to a cell were determined in separate experiments using radioactive reagents. In all experiments a challenge of $10^5$ or $10^6$ tumour cells, corresponding to 1000 and 10,000 times $LD_{100}$ were used. Attempts were made to characterize the type of immune response after challenge, using serological methods.

### MATERIALS AND METHODS

*Cells.*—The cell line STU-D17 used throughout was obtained from STU mouse embryo cells transformed by Rous sarcoma virus (Schmidt–Ruppin strain). The cell line was kindly supplied by Dr Heinz Bauer, University of Giessen, Germany.

*Radiochemicals.*—[$^{14}$C]-acetic anhydride, [$^{14}$C]-methylamine, [$^{14}$C]-dimethylsulphate, and N-[$^{14}$C]-ethylmaleimide were purchased from the Radiochemical Centre, Amersham, England, and [$^{14}$C]-formaldehyde from NEN, Boston, USA.

*Chemicals.* — 1-Ethyl-3(3-dimethylaminopropyl)-carbodiimide (EDC) was obtained from the Ott Chemical Co., Muskegon, Mich., USA. All other chemicals were of analytical grade, and were obtained from Merck, Darmstadt, Germany.

*Preparation of the cells.*—The cells were grown in minimum essential medium supplemented with 10% of foetal calf serum (Dulbecco and Freemann, 1959). The cells were harvested by treatment with trypsin, washed ×3 in ice-cold phosphate-buffered saline (PBS), and resuspended in PBS to give a standard concentration of $5 \times 10^6$ cells/ml.

*Conditions of cell modification.*—Generally, 10-ml portions of STU-D17 cells of standard concentration were reacted with the chemical reagents. In Table I the reaction conditions are listed of those modifying reagents which induced a significant immunogenic effect. In the case of modification with glutardialdehyde + butylamine both chemicals were added together. In the case of glutardialdehyde + EDC/methylamine the cells were washed after glutardialdehyde fixation, incubated in PBS for 18 h, followed by simultaneous addition of EDC and methylamine. Modification of cells was also performed with N-ethylmaleimide (50 mM), glutardialdehyde + methylamine (200 + 600 mM), glutardialdehyde + dimethylsulphate (200 + 50 mM), EDC + methylamine (2 + 150 mM) as well as by treatment with sodium peroxide (1 mM) followed by acetoacetylation with diketene (50 mM). Termination of the reactions was achieved by washing the modified cell samples 3× with cold PBS. The modified cells were injected into the animals without delay.

For the determination of the number of modifying groups per cell, 5-ml portions of STU-D17 cells were reacted with $^{14}$C-labelled chemicals.

*Immunization procedures.*—Highly inbred STU mice (Committee on Standardized Nomenclature for inbred strains of mice, 1968) 4–6 weeks old were used in all experiments. Groups of 10 animals were immunized, each with a single s.c. injection (0·2 ml) into the left flank, with doses varying between $10^2$ and $10^6$ of modified cells. Fourteen days after immunization, the mice received a challenge of $10^5$ or $10^6$ viable STU-D17 tumour cells (0·2 ml) s.c. into the neck. Controls were 20 animals

TABLE I.—*Reaction of STU-D17 Mouse Tumour Cells ($5 \times 10^6$ cells/ml) with Various Reagents in PBS pH 7·3*

| Reagent | Concentration (mM) | Reaction time (min) | Reaction temperature (°C) | % Cell killing |
|---|---|---|---|---|
| Dimethylsulphate[a] | 50 | 10 | 37 | 20 |
| Acetic anhydride | 50 | 10 | 4 | 30 |
| Diketene | 50 | 10 | 37 | 30 |
| Formaldehyde | 200 | 180[b] | 37 | 0–5 |
| Glutardialdehyde | 200 | 180[b] | 37 | 0–5 |
| Glutardialdehyde + butylamine | 200 +600 | 180[b] | 37 | 0–5 |
| Glutardialdehyde +(EDC+methylamine) | 200 +(2+150) | 180[b] +180 | 37 | 0–5 |

[a] Cells were suspended in PBS pH 7·3 containing 5% glycerol and 5% ethanol.
[b] After washing, the cells were further incubated in PBS pH 7·3 at room temperature for 18 h.

per group receiving only a challenge of viable tumour cells. When groups of mice were immunized with X-irradiated tumour cells, the cell samples were washed ×3 with cold PBS after exposure to 5000–6000 rad.

To study the influence of the interval between immunization and challenge, 3 groups of mice which had been immunized with tumour cells modified with formaldehyde received the challenge 42 days after immunization.

The effect of multiple immunization was studied with tumour cells modified with glutardialdehyde. The animals received 3 immunizations at weekly intervals, followed by challenge 2 weeks after the last immunization.

All samples of modified cells were tested for viability using groups of 10 mice. Each animal received an injection of $10^6$ modified cells in 0·2 ml PBS, and the appearance of tumours was observed over a period of 110 days.

*Serology.*—For comparative serological studies, the mice were bled 7 days after tumour challenge by puncturing the retro-orbital sinus. The individual sera of each group were pooled. Serum dilutions 1 : 20 were tested in a microcytotoxicity assay (Baldwin and Embleton, 1971) for cytotoxic antibodies directed against native STU-D17 cells. In addition, the sera were assayed in an indirect membrane fluorescence test (Beutner, Holborow and Johnson, 1967) using FITC-labelled rabbit anti-STU-immunoglobulin sera.

### RESULTS

One of the factors influencing the immunogenicity of an antigen has been shown to be the number of modifying groups per antigen molecule (Parish, 1971; Staab and Anderer, 1976). The immunogenic capacity to induce specific cellular immune responses increased with increasing numbers of modifying groups, but optimal effects were achieved before all the reactive sites of the antigen had reacted (Parish, 1971).

For all types of chemical modification performed with STU-D17 tumour cells, saturation curves have been established using $^{14}$C-labelled reagents. In the range of saturation of some reactions, a considerable portion of the cells was destroyed, most probably as a result of intensive chemical modification. Therefore the concentration of the reagents was reduced to give at most 30% loss of cells (see Table I). The ratio of modifying groups per cell could be calculated on the basis of the specific radioactivity of the reagents, the labelling of the cells and the cell number. The data are listed in Table II. In the case of the reactions

TABLE II.—*Ratios of Modifying Groups per Cell Given for Various Modification Reactions at Saturation and Standard Reaction Conditions*

| | Modifying groups/cell | |
|---|---|---|
| Modifying reagent | Saturation conditions | Standard conditions |
| [$^{14}$C]-dimethylsulphate | $1·8 \times 10^{10}$ | $6·8 \times 10^{9}$ |
| [$^{14}$C]-acetic anhydride | $4·2 \times 10^{10}$ | $2·1 \times 10^{10}$ |
| [$^{14}$C]-formaldehyde | $9·0 \times 10^{10}$ | $6·9 \times 10^{10}$ |
| Glutardialdehyde +[$^{14}$C]-methylamine | $7·1 \times 10^{10}$ | $6·2 \times 10^{10}$ |
| EDC+[$^{14}$C]-methylamine | $8·0 \times 10^{9}$ | $4·6 \times 10^{9}$ |
| N-[$^{14}$C]-ethylmaleimide | $1·2 \times 10^{10}$ | $5·1 \times 10^{9}$ |

with diketene, glutardialdehyde and butylamine, no radioactive label was used, but one can assume comparable ratios to those given for acetic anhydride, formaldehyde and methylamine respectively.

The STU-D17 tumour cells modified according to the standard conditions were routinely tested for viability *in vitro* by trypan blue uptake and *in vivo* by a single injection of $10^6$ modified cells per animal. No group of animals developed tumours within 110 days, except the group which received N-ethylmaleimide-modified tumour cells, where 70% of the animals showed tumour growth. The latter finding is in agreement with the results obtained with iodoacetate, which is another sulphydryl reagent (Jasmin, Piton and Rosenfeld, 1968). It is noteworthy that trypan blue uptake did not coincide with the loss of viability of the cells. In some cases only 20%

of the modified cells showed trypan blue uptake, but no tumours developed when the cells were injected into mice.

In the course of the immunization experiment we determined the number of animals which remained tumour-free during 90 days after challenge with viable cells. For those mice which developed tumours, the mean survival time was calculated as an additional criterion for induced immunity against the tumour transplant. In Table III the results of the experiments with modified cells are listed only for those which induced a significant immune reaction against the tumour challenge. The results of those experiments obtained with mice immunized with X-irradiated tumour cells are also given.

When a challenge of $10^5$ tumour cells was given, in 2 cases up to 40% of the animals showed full protection and remained tumour-free, whereas the experimental groups of mice immunized with

TABLE III.—*Protection of STU Mice against Challenge with* $10^5$ *or* $10^6$ *STU-D17 Tumour Cells after Pretreatment with Various Dosages of Modified STU-D17 Tumour Cells. The Challenge was given* 14 *Days after Immunization Unless Otherwise Stated*

| Type of modification | Immunization log cells per animal | Challenge log cells | Tumour-free animals[a] % per group | Mean survival[b] Days | % of control |
|---|---|---|---|---|---|
| Controls | — | 5 | 0 | 40 | 100 |
| | — | 6 | 0 | 24 | 100 |
| Dimethylsulphate | 2 | 5 | 20 | 49 | 123 |
| | 3 | 5 | 0 | 57 | 142 |
| | 4 | 5 | 30 | 73 | 183 |
| | 6 | 5 | 30 | 39 | 98 |
| | 2 | 6 | 0 | 27 | 112 |
| | 3 | 6 | 30 | 28 | 117 |
| | 4 | 6 | 30 | 61 | 254 |
| | 6 | 6 | 0 | 32 | 133 |
| Acetic anhydride | 2 | 5 | 20 | 43 | 108 |
| | 3 | 5 | 40 | 72 | 180 |
| | 4 | 5 | 20 | 55 | 138 |
| | 6 | 5 | 20 | 42 | 105 |
| Diketene | 2 | 5 | 10 | 41 | 103 |
| | 3 | 5 | 10 | 52 | 130 |
| | 4 | 5 | 10 | 42 | 105 |
| | 6 | 5 | 10 | 38 | 95 |
| | 2 | 6 | 0 | 40 | 167 |
| | 3 | 6 | 0 | 41 | 171 |
| | 4 | 6 | 10 | 40 | 167 |
| | 6 | 6 | 0 | 36 | 150 |
| Formaldehyde | 2 | 5 | 0 | 43 | 108 |
| | 3 | 5 | 30 | 50 | 125 |
| | 4 | 5 | 10 | 62 | 155 |
| | 6 | 5 | 10 | 50 | 125 |
| | 2[c] | 5 | 0 | 45 | 113 |
| | 4[c] | 5 | 0 | 50 | 125 |
| | 6[c] | 5 | 0 | 50 | 125 |
| EDC+methylamine | 2 | 5 | 0 | 53 | 132 |
| | 3 | 5 | 0 | 61 | 153 |
| | 4 | 5 | 10 | 45 | 113 |
| | 6 | 5 | 0 | 45 | 113 |
| Glutardialdehyde | 2 | 5 | 0 | 42 | 105 |
| | 3 | 5 | 10 | 45 | 113 |
| | 4 | 5 | 0 | 39 | 98 |
| | 6 | 5 | 0 | 38 | 95 |
| | 2[d] | 5 | 0 | 48 | 120 |
| | 4[d] | 5 | 20 | 52 | 130 |
| | 6[d] | 5 | 0 | 48 | 120 |

TABLE III.—*Continued.*

| Type of modification | Immunization log cells per animal | Challenge log cells | Tumour-free[a] animals % per group | Mean survival[b] Days | % of control |
|---|---|---|---|---|---|
| Glutardialdehyde +butylamine | 2 | 5 | 30 | 46 | 115 |
| | 3 | 5 | 0 | 42 | 105 |
| | 4 | 5 | 20 | 47 | 118 |
| | 6 | 5 | 10 | 51 | 128 |
| Glutardialdehyde +(EDC+methylamine) | 2 | 5 | 40 | 67 | 168 |
| | 3 | 5 | 30 | 56 | 140 |
| | 4 | 5 | 30 | 51 | 128 |
| | 6 | 5 | 0 | 28 | 70 |
| | 2 | 6 | 0 | 28 | 117 |
| | 3 | 6 | 0 | 22 | 92 |
| | 4 | 6 | 20 | 22 | 92 |
| | 6 | 6 | 20 | 25 | 104 |
| X-irradiation | 2 | 5 | 20 | 50 | 125 |
| | 3 | 5 | 20 | 59 | 148 |
| | 4 | 5 | 0 | 58 | 145 |
| | 6 | 5 | 0 | 57 | 142 |
| | 2 | 6 | 0 | 27 | 112 |
| | 3 | 6 | 0 | 23 | 96 |
| | 4 | 6 | 0 | 26 | 108 |
| | 6 | 6 | 0 | 27 | 112 |

[a] No palpable tumours 90 days after challenge.
[b] Tumour-bearing animals only.
[c] Challenge 42 days after immunization.
[d] 3 immunizations at weekly intervals.

X-irradiated tumour cells showed at most up to 20% of tumour-free animals. Most of the animals which developed tumours showed highly increased mean survival times. The maximum mean survival times were usually found in those groups which also had the highest percentage of tumour-free animals. It was found that these maxima were essentially dependent on the number of modified cells used for immunization. As can be seen from Table III, the best results were obtained with an immunizing dose of between $10^2$ and $10^4$ modified cells. Modification with dimethylsulphate and acetic anhydride as well as complex modification with glutardialdehyde/(EDC + methylamine) induced an almost equal increase in the immunogenicity of the STU-D17 cells. These reactions also showed the optimal effect which had been found in the course of our work. All the other modified cell samples, including the X-irradiated cells, proved to be less efficient in inducing an immune response, but still showed significant tumour protection and increased mean survival times.

Moreover, in the case of immunizations with cell samples modified with dimethylsulphate, diketene or glutardialdehyde/(EDC + methylamine) and with X-irradiated cells, the challenge dosage was raised to $10^6$ tumour cells. Tumour protection and increased mean survival time induced by dimethylsulphate-modified cells were as efficient as with the challenge of $10^5$ tumour cells. In the other cases a significant but lower tumour protection was observed (Table III).

In order to obtain some information on the persistence of tumour protection, in one experiment animals immunized with formaldehyde-treated cells received $10^5$ viable tumour cells 42 days instead of 14 days after immunization. As can be seen from Table III, no tumour-free animals and only a moderate increase in the mean survival time were observed.

The effect of multiple immunization was studied with glutardialdehyde-treated cells, which induced only weak immunogenicity after a single immunizing injection. In this experiment, 3 injections at weekly intervals were given, followed by a challenge of $10^5$ tumour cells 14 days later. Only a slight increase in tumour protection and in the mean survival time were obtained.

The persistence of tumour immunity after immunization and challenge was studied with tumour-free animals which had been immunized with glutardialdehyde/(EDC + methylamine) modified cells. A second challenge of $10^5$ tumour cells was given 180 days after the first challenge. Ninety days later 80% of the animals were still tumour-free.

All the other groups immunized with modified cell samples but not listed in Table III had only up to 20% tumour-free animals, and the mean survival times were only slightly increased. As already mentioned, cell samples modified with N-ethylmaleimide still had the capacity to induce tumours in 70% of the animals when the immunizing dosage was $10^6$ cells. The other 30% of the animals remained tumour-free, but did not show any tumour protection when a challenge of $10^5$ viable tumour cells was given. When immunization was done with doses of $10^3$ or $10^4$ modified cells, no tumour growth was observed, but the mean survival time after challenge with viable tumour cells was distinctly less than that of the control groups.

To characterize the type of immune response induced by the modified cell samples, the pooled sera obtained 7 days after the tumour challenge were assessed in the microcytotoxicity assay, and in the indirect membrane immunofluorescence test. With the exception of the animals which had been immunized with cells modified with N-ethylmaleimide or by treatment with $Na_2O_2$/diketene, no antibodies (*i.e.* less than 50% cytotoxicity) could be detected in the sera of all the other groups with either method. The detection of antibodies in the sera of animals immunized with N-ethylmaleimide-treated cells was dependent on the immunizing dose. Antibodies were present when the animals were immunized with $10^3$, $10^4$ and $10^6$ modified cells (cytotoxicity: 96%, 93% and 94% respectively). The dosage of $10^2$ modified cells did not induce detectable amounts of humoral antibodies (50% cytotoxicity). The same holds true for animals immunized with $Na_2O_2$/diketene-treated cells. Cytotoxicity was 52%, 60% and 77% when the immunizing dosage was $10^3$, $10^4$ or $10^6$ modified cells. These findings correlate with the partly decreased mean survival times in these groups, indicating that the antibodies might have exhibited an enhancing effect on tumour growth.

### DISCUSSION

For the interpretation of any effect on the immunogenicity of tumour cells which can be induced by chemically distinct modifying groups, it is of great importance that the antigenic patterns of tumour cells and experimental animals differ by nothing but tumour antigens. To meet these requirements we chose a syngeneic tumour cell line, thus excluding any effects due to differences in histocompatibility antigens.

On the other hand we thought it advantageous to work with modifying groups which were known to have no detectable haptenic character. With one exception, all modifying reactions with tumour cells as well as X-irradiation were carried on to the extent that the modified cells were no longer capable of cell division. The effects on the immunogenicity of the tumour cells were characterized by two parameters: tumour protection and mean survival time of tumour-bearing animals. Tumour protection was established against a tumour challenge of 1000 or 10,000 times the $LD_{100}$. Full tumour protection exceeding 90 days was thought to be sufficient, since this period is analogous to more than 5 years of human life.

Optimal tumour protection and increased mean survival time were found to be dependent on the modifying groups and on the dose of modified cells used for immunization. Modification of tumour cells with dimethylsulphate or acetic anhydride, or of glutardialdehyde-fixed cells treated with carbodiimide and methylamine, induced the most efficient alteration of immunogenicity. Best results were found with the lower immunizing doses rather than with the high dose of $10^6$ modified cells. In some experiments even $10^2$ modified cells were sufficient to induce a significant tumour protection. This can be understood in terms of a critical range of the amount of antigen responsible for antigen recognition. Since in the experiments showing optimal tumour protection no humoral antibodies could be detected, one can conclude that this critical range only involves the cellular immune response.

It is noteworthy that several types of chemical modification of the tumour cells chosen for our study proved to be more efficient in inducing tumour protection than X-irradiation. The radiation-induced alterations of immunogenicity are compatible with the findings of other authors who had investigated leukaemic mouse cells (Lin, Huber and Murphy, 1969).

In our study we selected modifying groups which were aliphatic, of small size and, when conjugated with proteins, were known to induce cellular immunity against protein antigens without inducing humoral antibodies against the modifying groups (Parish, 1971; Coon and Hunter, 1972; Thompson, Harries and Benjamini, 1972; Staab and Anderer, 1976; Benacerraf and Gell, 1959). The number of modifying groups per cell differed from saturation by a factor of 2 at most, thus following the findings obtained with protein antigens (Parish, 1971; Staab and Anderer, 1976). Considering the membrane structure and the type of chemical reagents used, one has to assume that the modifying groups had reacted with sites not only on the cell surface but also inside the cell. The chemical alterations on the cell surface induced by the modifying groups which led to an efficient increase in the capacity to induce a cellular immune response, can be generally correlated with changes of the charge pattern on the cell surface. All the modifying reactions investigated resulted in an overall reduction of charges. Dimethylsulphate reacts with amino groups without a change in the charge, and with SH-groups and phenolic OH-groups, but also with carboxylic groups (Staab and Anderer, 1976) thus reducing the total number of negative charges. The reaction of EDC and methylamine also diminishes the number of negative charges. A reduction of positive charges results from the reactions with acetic anhydride, diketene and with aldehydes.

On the basis of our results one has to question whether alterations of the surface charge pattern (*i.e.* reduction of negative and positive charges) do generally increase the immunogenic capacity by promoting a cellular immune response. It would be, therefore, of great interest to investigate the modification reactions which have proved to be efficient in our study in other syngeneic systems of tumour cells and experimental animals.

The authors would like to thank Ms U. Abrutsch for her excellent technical assistance.

### REFERENCES

BALDWIN, R. W. & EMBLETON, M. J. (1971) Demonstration by Colony Inhibition Methods of Cellular and Humoral Immune Reactions to Tumour-specific Antigens Associated with Aminoazo-dye-induced Rat Hepatomas. *Int. J. Cancer*, **7**, 17.

BENACERRAF, B. & GELL, P. G. H. (1959) Studies on Hypersensitivity: I. Delayed and Arthus-type Skin Reactivity to Protein Conjugates in Guinea Pigs. *Immunology*, **2**, 53.

BEUTNER, E. H., HOLBOROW, E. I. & JOHNSON, O. (1967) Quantitative Studies of Immunofluorescent Staining: I. Analysis of Mixed Immunofluorescence. *Immunology*, **12**, 327.

COMMITTEE ON STANDARDIZED NOMENCLATURE FOR INBRED STRAINS OF MICE (1968) Standardized Nomenclature for Inbred Strains of Mice: Fourth Listing. *Cancer Res.*, **28**, 391.

COON, J. & HUNTER, R. (1972) Selective Induction of Delayed Hypersensitivity by a Lipid Conjugated Protein Antigen which is Localized in Thymus Dependent Lymphoid Tissue. *J. Immunol.*, **110**, 183.

DULBECCO, R. & FREEMANN, C. (1959) Plaque Production by the Polyoma Virus. *Virology*, **8**, 396.

JASMIN, C., PITON, C. & ROSENFELD, C. (1968) Effets de l'Iodoacétamide sur les Cellules de la Leucémie Virale de Rauscher. *Int. J. Cancer*, **3**, 254.

LIN, J. S. L., HUBER, N. & MURPHY, W. H. (1969) Immunization of C58 Mice to Line $I_b$ Leukemia. *Cancer Res.*, **29**, 2157.

PANTELEAKIS, P. N., LARSON, V. M., MCALEER, W. J. & HILLEMANN, M. R. (1971) Variations in Immunogenicity of Disrupted Cells Prepared from an Adenovirus-7 Hamster Tumour Cell Line. *J. natn. Cancer Inst.*, **46**, 1195.

PARISH, C. R. (1971) Immune Response to Chemically Modified Flagellin: II. Evidence for a Fundamental Relationship between Humoral and Cell-mediated Immunity. *J. exp. Med.*, **134**, 21.

PRAGER, M. D., DERR, J., SWANN, A. & COTROPIA, J. (1971) Immunization with Chemically Modified Lymphoma Cells. *Cancer Res.*, **31**, 1488.

PRAGER, M. D. & BAECHTEL, F. S. (1973) Methods for Modification of Cancer Cells to Enhance their Antigenicity. In *Methods in Cancer Research*. Ed. Harris Busch, Vol. **9**. New York: Academic Press, p. 339.

STAAB, H. J. & ANDERER, F. A. (1976) Structure and Immunogenic Behaviour of Methylated Tobacco Mosaic Virus. *Biochim. biophys. Acta*, **427**, 453.

THOMPSON, K., HARRIES, M. & BENJAMINI, E. (1972) Cellular and Humoral Immunity: A Distinction in Antigenic Recognition. *Nature, New Biol.*, **238**, 20.

YOSHIMURA, M. & KABURAKI, T. (1963) Immunization against Ehrlich Mouse Ascites Carcinoma with Chemically Devitalized Tumour Cells. *Jap. J. Pharm.*, **13**, 127.